# Pregnancy and Labor Complications in Female Survivors of Childhood Cancer: The British Childhood Cancer Survivor Study

**DOI:** 10.1093/jnci/djx056

**Published:** 2017-04-17

**Authors:** Raoul C. Reulen, Chloe J. Bright, David L. Winter, Miranda M. Fidler, Kwok Wong, Joyeeta Guha, Julie S. Kelly, Clare Frobisher, Angela B. Edgar, Roderick Skinner, W. Hamish B. Wallace, Mike M. Hawkins

**Affiliations:** **Affiliations of authors:** Centre for Childhood Cancer Survivor Studies, Institute of Applied Health Research, Robert Aitken Building, University of Birmingham, Edgbaston, Birmingham, UK (RCR, CJB, DLW, MMF, KW, JG, JSK, CF, MMH); Department of Paediatric Haematology and Oncology, Royal Hospital for Sick Children, University of Edinburgh, Edinburgh, UK (ABE, WHBW); Department of Paediatric and Adolescent Haematology and Oncology, and Children’s BMT Unit, Great North Children’s Hospital, Royal Victoria Infirmary, Newcastle upon Tyne, UK (RS).

## Abstract

**Background:** Female survivors of childhood cancer treated with abdominal radiotherapy who manage to conceive are at risk of delivering premature and low-birthweight offspring, but little is known about whether abdominal radiotherapy may also be associated with additional complications during pregnancy and labor. We investigated the risk of developing pregnancy and labor complications among female survivors of childhood cancer in the British Childhood Cancer Survivor Study (BCCSS).

**Methods:** Pregnancy and labor complications were identified by linking the BCCSS cohort (n = 17 980) to the Hospital Episode Statistics (HES) for England. Relative risks (RRs) of pregnancy and labor complications were calculated by site of radiotherapy treatment (none/abdominal/cranial/other) and other cancer-related factors using log-binomial regression. All statistical tests were two-sided.

**Results:** A total of 2783 singleton pregnancies among 1712 female survivors of childhood cancer were identified in HES. Wilms tumor survivors treated with abdominal radiotherapy were at threefold risk of hypertension complicating pregnancy (relative risk = 3.29, 95% confidence interval [CI] = 2.29 to 4.71), while all survivors treated with abdominal radiotherapy were at risk of gestational diabetes mellitus (RR = 3.35, 95% CI = 1.41 to 7.93) and anemia complicating pregnancy (RR = 2.10, 95% CI = 1.27 to 3.46) compared with survivors treated without radiotherapy. Survivors treated without radiotherapy had similar risks of pregnancy and labor complications as the general population, except survivors were more likely to opt for an elective cesarean section (RR = 1.39, 95% CI = 1.16 to 1.70).

**Conclusions:** Treatment with abdominal radiotherapy increases the risk of developing hypertension complicating pregnancy in Wilms tumor survivors, and diabetes mellitus and anemia complicating pregnancy in all survivors. These patients may require extra vigilance during pregnancy.

Survival from childhood cancer has improved considerably over the last few decades, and currently nearly 80% of children diagnosed with cancer survive at least five years (1). This dramatic improvement in survival is mainly attributable to advances in multimodality therapy with combination chemotherapy and improved radiotherapy delivery. However, various studies have shown that previous cancer treatment, particularly following radiotherapy, increases the long-term risk of developing adverse health outcomes, including second primary cancer, cardiovascular disease, infertility, and adverse pregnancy outcomes (2,3).

Female survivors of childhood cancer who have been treated with abdominal radiotherapy and who subsequently managed to conceive are at risk of premature delivery and delivering low-birthweight offspring (4–10). The exact mechanism underlying this risk is not entirely clear, but it has been postulated that exposure to abdominal irradiation increases myometrial fibrosis and negatively affects vascular and/or muscular development of the uterus (11,12). Although the risks of premature delivery and delivering low-birthweight offspring in survivors of childhood cancer are well documented (4–10), it is unclear whether previous treatment with abdominal radiotherapy for childhood cancer may also be associated with additional complications during pregnancy and labor. Green et al. (5,13) reported that female survivors of unilateral Wilms’ tumor treated with flank irradiation are at risk of fetal malposition, early or threatened labor, and developing hypertension complicating pregnancy, but to the best of our knowledge, no other large-scale study has investigated the risk of specific pregnancy and labor complications in women treated with abdominal radiotherapy for childhood cancer.

The principal aim of this study was to investigate the risks of developing pregnancy and labor complications ascertained through linkage with hospital electronic health records in female survivors treated with abdominal radiotherapy for childhood cancer.

## Methods

### British Childhood Cancer Survivor Study

The British Childhood Cancer Survivor Study is a large-scale population-based cohort of 17 980 five-year survivors of childhood cancer who were diagnosed with cancer between age 0 to 14 years from 1940 to 1991 in Britain (14). The cohort was ascertained through the National Registry of Childhood Tumours. The BCCSS cohort was linked to the population-based Hospital Episode Statistics (HES) for England—an electronic database that records data on patients’ hospital admissions (inpatient), outpatient appointments, and accident and emergency attendances at National Health Service (NHS) or private hospitals (if care was commissioned by the NHS). HES is managed by NHS digital and developed as a system for hospitals to get paid for administered patient care. For this study, the BCCSS cohort was linked to the inpatient HES data set from April 1, 1997, to December 31, 2012, by a third party (Northgate Solutions) using NHS number, date of birth, and postcode. Approval to link the BCCSS cohort to HES without prior individual patient consent was obtained from the Confidentiality Advisory Group and the National Research Ethics Service.

As HES only covers England, 2116 (11.8%) five-year survivors who were alive and residing in Scotland or Wales were excluded from analyses. Among the 6192 female survivors in the cohort who were alive as of April 1, 1997, 5126 (82.8%) had at least one recorded hospital admission in the HES inpatient data set.

The HES inpatient data set includes any records relating to care given for births that took place in NHS hospitals. Although home deliveries and births in private hospitals should be recorded in HES, practically few are (15), and hence these deliveries were excluded (n = 35). Pregnancies that resulted in a multiple birth (n = 85) were also excluded as the risk of pregnancy and labor outcomes is likely to differ from singleton births.

For comparisons with the general population, a random sample of 25 000 deliveries was extracted from the entire (anonymized) HES database (n = 8 821 531 deliveries). A sample of 25 000 was decided upon because at that size the sample was sufficiently large to provide ample statistical power, and, moreover, larger samples (eg, n = 100 000) resulted in similar results (results not shown).

### Pregnancy and Labor Complications

Pregnancy and labor complications were only evaluated if there were at least 50 affected pregnancies. Medical conditions and complications are recorded in HES using the *International Classification of Diseases* (ICD), revision 10. Specifically, we investigated the risk of: hypertension complicating pregnancy (ICD-10: O10-O11/O13-O16), gestational diabetes mellitus (O24.4), anemia complicating pregnancy (O99.0), malpresentation of fetus (O32), maternal care due to uterine scar from previous surgery (O34.2), fetal problems (O36), premature rupture of membranes (ie, rupture of the amniotic sac; O42), prolonged pregnancy (O48), abnormalities of forces of labor (O62), long labor (O63), obstructed labor due to malposition of fetus (O64), delivery complicated by fetal stress (O68), umbilical cord complications (O69), perineal laceration (O70), and postpartum hemorrhage (O72). In addition, the likelihood of the delivery method being elective or emergency cesarean and whether there were any high-risk pregnancies that needed supervision (ICD-10: Z35) was also evaluated. Adverse pregnancy outcomes evaluated in this study included low-birthweight baby, preterm delivery, and stillbirth.

### Statistical Analysis

A log-binomial regression model—with a population-averaged generalized estimating equation modification accounting for correlation between pregnancies of the same survivor—was used to calculate relative risk (RR) estimates of pregnancy and labor complications by type of childhood cancer, site of radiotherapy treatment (none/cranial/abdominal/other), age at childhood cancer, and calendar period of treatment (<1980/1980–1984/1985–1991). Abdominal radiotherapy was defined as any radiotherapy received for a tumor in the abdomen or pelvic volume. All models included the potential confounders maternal age and parity unless otherwise specified. Relative risks were also calculated for Wilms tumor survivors as these would have received some of the highest doses of abdominal irradiation and also comprise a sufficiently large group to consider separately. To determine whether survivors differed in their risk of developing pregnancy and labor complications from the general population independent of an effect of radiotherapy, survivors treated without radiotherapy were chosen for comparisons with the general population.

Although the completeness of HES ascertainment increased over calendar years, not all deliveries may have been recorded within HES, particularly before 2002 (15,16). A recent exercise whereby HES delivery records from 2002 to 2010 were linked with the Office of National Statistics (ONS) birth registrations found that 96.8% of all births recorded by ONS were also recorded in HES, suggesting that—at least for the period 2002 to 2010—the majority of deliveries are captured by HES (17). To ascertain whether potential underreporting of deliveries and adverse pregnancy and labor outcomes in the years 1997 to 2001 could have potentially introduced bias, we conducted a sensitivity analysis by fitting HES calendar year (1997–2001 vs 2002–2012) as an interaction term in our model. Such an interaction term should detect whether there is statistically significant variation in the risk of developing a specific pregnancy or labor complication by HES calendar year.

For factors with multiple categories, a test for homogeneity of the relative risks across the levels of the factor was also performed, and *P*_heterogeneity_ was used to indicate the statistical significance of the overall test. All analyses were performed using Stata 14 (StataCorp, College Station, Texas) (18). Statistical significance was defined at a two-sided *P* value of less than .05. 

## Results

### Cohort Characteristics

A total of 2783 singleton deliveries among 1712 female survivors of childhood cancer were available for analysis (Table 1). The mean maternal age at the delivery-related admission was 28.7 years (SD = 5.4 years). The majority of recorded deliveries were among survivors of leukemia (32.9%, n = 915), central nervous system tumor (12.1%, n = 336), and Wilms tumor (11.6%, n = 322). In terms of site of radiotherapy treatment, 205 (12.0%) survivors had received abdominal radiotherapy (of whom 127, 62.0%, were Wilms tumor survivors), 543 (31.7%) had received radiotherapy to the brain, 529 (30.9%) had not been treated with any radiotherapy, and 146 (8.5%) had received radiotherapy to sites other than the abdomen or brain.

**Table 1.** Characteristics of female survivors with at least one recorded delivery episode in Hospital Episode Statistics (HES; n = 1712) and singleton pregnancies of female survivors recorded in HES (n = 2783)

Characteristic	Individuals (n = 1712) No. (%)	Pregnancies (n = 2783) No. (%)	Excluded pregnancies (n = 120)* No. (%)
Type of childhood cancer			
Leukemia	546 (31.9)	915 (32.9)	32 (26.7)
Hodgkin lymphoma	93 (5.4)	153 (5.5)	11 (9.2)
Non-Hodgkin lymphoma	69 (4.0)	111 (4.0)	5 (4.2)
CNS tumor	218 (12.7)	336 (12.1)	19 (15.8)
Neuroblastoma	85 (5.0)	135 (4.9)	5 (4.2)
NH-retinoblastoma	89 (5.2)	143 (5.1)	3 (2.5)
H-retinoblastoma	35 (2.0)	58 (2.1)	5 (4.2)
Wilms tumor	199 (11.6)	322 (11.6)	14 (11.7)
Bone tumor	92 (5.4)	145 (5.2)	5 (4.2)
Soft-tissue sarcoma	112 (6.5)	185 (6.6)	6 (5.0)
Other	174 (10.2)	280 (10.1)	15 (12.5)
Type of radiotherapy			
None	529 (30.9)	860 (30.9)	44 (36.7)
Brain	543 (31.7)	901 (32.4)	29 (24.2)
Other (nonbrain/abdominal)	146 (8.5)	231 (8.3)	12 (10.0)
Abdominal	205 (12.0)	326 (11.7)	9 (7.5)
Abdominal after:			
Wilms tumor	127 (7.4)	207 (7.4)	3 (2.5)
Hodgkin lymphoma	19 (1.1)	30 (1.1)	2 (1.7)
Soft-tissue sarcoma	16 (0.9)	24 (0.9)	1 (0.8)
Bone tumor	15 (0.9)	23 (0.8)	1 (0.8)
Non-Hodgkin lymphoma	9 (0.5)	15 (0.5)	1 (0.8)
Neuroblastoma	7 (0.4)	10 (0.4)	0 (0.0)
Other	12 (0.7)	17 (0.6)	1 (0.8)
Unknown	289 (16.9)	465 (16.7)	26 (21.7)
Wilms tumor			
No radiotherapy	59 (3.4)	96 (3.4)	4 (3.3)
Abdominal radiotherapy	127 (7.4)	207 (7.4)	3 (2.5)
Unknown	13 (0.8)	19 (0.7)	7 (5.8)
Age at childhood cancer diagnosis, y			
0–4	802 (46.8)	1315 (47.3)	39 (32.5)
5–9	481 (28.1)	781 (28.1)	49 (40.8)
10–14	429 (25.1)	687 (24.7)	32 (26.7)
Calendar year of childhood cancer			
1955–1969	78 (4.6)	102 (3.7)	3 (2.5)
1970–1974	175 (10.2)	246 (8.8)	13 (10.8)
1975–1979	308 (18.0)	500 (18.0)	21 (17.5)
1980–1984	465 (27.2)	809 (29.1)	42 (35.0)
1985–1991	686 (40.1)	1126 (40.5)	41 (34.2)
Maternal age, y			
<18	32 (1.9)†	33 (1.2)	0 (0.0)
18–24	467 (27.3)†	636 (22.9)	29 (24.2)
25–29	532 (31.1)†	873 (31.4)	38 (31.7)
30–34	457 (26.7)†	818 (29.4)	33 (27.5)
≥35	224 (13.1)†	423 (15.2)	20 (16.7)

* Home deliveries and births in private hospitals (n = 35), as well as pregnancies that resulted in a multiple birth (n = 85), were excluded. CNS = central nervous system; H = heritable; HES = Hospital Episode Statistics; NH = nonheritable.

† Relates to first recorded delivery episode.

### Hypertension, Diabetes Mellitus, and Anemia in Pregnancy

The relative risk of developing hypertension complicating pregnancy was substantially elevated among Wilms tumor survivors treated with abdominal radiotherapy (RR = 3.29, 95% confidence interval [CI] = 2.29 to 4.71) vs those treated without radiotherapy (Table 2). Twenty-three point seven percent of all Wilms tumor survivors treated with abdominal radiotherapy experienced hypertension that complicated the pregnancy vs only 7.1% of all survivors not treated with radiotherapy and 6.0% of women in the general population. Wilms tumor survivors not treated with abdominal radiotherapy were, however, not at statistically significant risk of developing hypertension complicating pregnancy (RR = 1.36, 95% CI = 0.68 to 2.71). Other survivors (ie, non-Wilms tumor) treated with abdominal radiotherapy were not at statistically significant risk either (RR = 1.09, 95% CI = 0.52 to 2.28). Similar relative risks as reported above were observed when excluding preexisting hypertension (ICD10: O10-O11), that is, for gestational hypertension only (Table 2).

**Table 2.** Relative risk of developing a hypertensive disorder, diabetes mellitus, or anemia in pregnancy among female survivors of childhood cancer

Characteristic	Maternal disorders related to pregnancy									Other obstetric conditions		
	Hypertension complicating pregnancy* ICD10: O10-O11, O13-O16			Hypertension complicating pregnancy* (excluding preexisting hypertension) ICD10:O13-O16			Gestational diabetes mellitus*,† ICD10: O24.4			Anemia complicating pregnancy*,‡ ICD10: O99.0		
	No. (%)	RR (95% CI)	*P*§	No. (%)	RR (95% CI)	*P*§	No. (%)	RR (95% CI)	*P*§	No. (%)	RR (95% CI)	*P*§
General population	1495 (6.0)	1.00 (ref.)		1013 (4.1)	1.00 (ref.)		390 (1.6)	1.00 (ref.)		1099 (4.4)	1.00 (ref.)	
Female survivors*‖*	61 (7.1)	1.23 (0.95 to 1.60)	.11	40 (4.7)	1.18 (0.85 to 1.65)	.32	13 (1.5)	0.91 (0.49 to 1.71)	.72	34 (4.0)	0.88 (0.58 to 1.29)	.52
Type of childhood cancer												
Leukemia	81 (8.9)	1.00 (ref.)		51 (5.6)	1.00 (ref.)		18 (2.0)	1.00 (ref.)		52 (5.7)	1.00 (ref.)	
Hodgkin lymphoma	10 (6.5)	0.62 (0.30 to 1.25)	.18	6 (3.9)	0.61 (0.23 to 1.58)	.31	5 (3.3)	1.33 (0.46 to 3.90)	.60	10 (6.5)	1.12 (0.59 to 2.13)	.73
Non-Hodgkin lymphoma	13 (11.7)	1.18 (0.62 to 2.25)	.61	9 (8.1)	1.34 (0.61 to 2.98)	.47	4 (3.6)	1.64 (0.49 to 5.56)	.42	8 (7.2)	1.18 (0.56 to 2.51)	.66
CNS tumor	25 (7.4)	0.83 (0.53 to 1.30)	.41	15 (4.5)	0.81 (0.45 to 1.45)	.47	4 (1.2)	0.60 (0.21 to 1.74)	.35	17 (5.1)	0.86 (0.48 to 1.54)	.61
Neuroblastoma	17 (12.6)	1.41 (0.80 to 2.48)	.24	13 (9.6)	1.71 (0.90 to 3.23)	.10	2 (1.5)	0.99 (0.23 to 4.32)	.99	4 (3.0)	0.49 (0.18 to 1.32)	.16
NH-retinoblastoma	6 (4.2)	0.50 (0.22 to 1.14)	.10	2 (1.4)	0.27 (0.07 to 1.11)	.07	4 (2.8)	1.24 (0.37 to 4.13)	.73	3 (2.1)	0.38 (0.12 to 1.18)	.09
H-retinoblastoma	3 (5.2)	0.66 (0.22 to 2.01)	.46	3 (5.2)	1.05 (0.34 to 3.26)	.93	0 (0.0)	—	—	3 (5.2)	0.94 (0.30 to 2.91)	.91
Wilms tumor	61 (18.9)	2.12 (1.52 to 2.97)	<.001	44 (13.7)	2.38 (1.56 to 3.64)	<.001	8 (2.5)	1.27 (0.49 to 3.25)	.62	21 (6.5)	1.15 (0.70 to 1.89)	.57
Bone tumor	9 (6.2)	0.64 (0.31 to 1.33)	.23	7 (4.8)	0.76 (0.32 to 1.81)	.53	1 (0.7)	0.39 (0.05 to 2.91)	.36	7 (4.8)	0.82 (0.32 to 2.06)	.67
Soft-tissue sarcoma	16 (8.6)	0.93 (0.53 to 1.64)	.80	12 (6.5)	1.13 (0.58 to 2.18)	.72	5 (2.7)	1.11 (0.30 to 4.01)	.88	10 (5.4)	0.95 (0.47 to 1.93)	.88
Other	21 (7.5)	0.85 (0.52 to 1.40)	.54	14 (5.0)	0.91 (0.49 to 1.67)	.76	5 (1.8)	0.83 (0.30 to 2.34)	.73	8 (2.9)	0.51 (0.24 to 1.06)	.07
*P*_heterogeneity_§			<.001			<.001			.93			.47
Treated with radiotherapy												
No	61 (7.1)	1.00 (ref.)		40 (4.7)	1.00 (ref.)		13 (1.5)	1.00 (ref.)		34 (4.0)	1.00 (ref.)	
Brain	82 (9.1)	1.25 (0.89 to 1.76)	.20	49 (5.4)	1.17 (0.75 to 1.82)	.49	19 (2.1)	1.61 (0.72 to 3.59)	.24	49 (5.4)	1.39 (0.89 to 2.18)	.15
Other (nonbrain/abdominal)	22 (9.5)	1.28 (0.76 to 2.14)	.36	18 (7.8)	1.64 (0.89 to 3.02)	.11	5 (2.2)	1.61 (0.55 to 4.67)	.38	5 (2.2)	0.57 (0.22 to 1.44)	.23
Abdominal	59 (18.1)	2.43 (1.70 to 3.47)	<.001	42 (12.9)	2.69 (1.72 to 4.22)	<.001	16 (4.9)	3.35 (1.41 to 7.93)	.01	27 (8.3)	2.10 (1.27 to 3.46)	.004
Abdominal non-Wilms	10 (8.4)	1.09 (0.52 to 2.28)	.81	7 (5.9)	1.17 (0.48 to 2.83)	.73	8 (6.7)	4.27 (1.54 to 11.83)	.01	11 (9.2)	2.25 (1.13 to 4.49)	.02
Abdominal Wilms only	49 (23.7)	3.29 (2.29 to 4.71)	<.001	35 (16.9)	3.59 (2.27 to 5.68)	<.001	8 (3.9)	2.73 (1.00 to 7.62)	.05	16 (7.7)	2.00 (1.13 to 3.57)	.02
No radiotherapy Wilms only	9 (9.4)	1.36 (0.68 to 2.71)	.38	7 (7.3)	1.60 (0.70 to 3.64)	.26	0 (0.0)	—	—	4 (4.2)	1.05 (0.36 to 3.12)	.93
*P*_heterogeneity_§			<.001			<.001			.23			.01
Age at diagnosis, y												
0–4	136 (10.3)	1.00 (ref.)		93 (7.1)	1.00 (ref.)		31 (2.4)	1.00 (ref.)		74 (5.6)	1.00 (ref.)	
5–9	70 (9.0)	0.96 (0.69 to 1.34)	.83	46 (5.9)	0.86 (0.56 to 1.32)	.48	13 (1.7)	0.61 (0.28 to 1.34)	.22	40 (5.1)	0.75 (0.46 to 1.24)	.27
10–14	56 (8.2)	1.06 (0.68 to 1.64)	.78	37 (5.4)	1.03 (0.60 to 1.79)	.91	12 (1.7)	0.81 (0.36 to 1.80)	.61	29 (4.2)	0.67 (0.38 to 1.19)	.17
*P*_trend_§			.85			.94			.54			.15
Decade of diagnosis												
<1980	86 (10.1)	1.00 (ref.)		54 (6.4)	1.00 (ref.)		14 (1.7)	1.00 (ref.)		49 (5.8)	1.00 (ref.)	
1980–1984	73 (9.0)	1.00 (0.70 to 1.43)	.99	50 (6.2)	1.06 (0.67 to 1.67)	.80	19 (2.3)	1.53 (0.61 to 3.83)	.36	41 (5.1)	0.82 (0.50 to 1.36)	.44
1985–1991	92 (9.3)	1.05 (0.66 to 1.66)	.85	65 (6.6)	1.16 (0.66 to 2.03)	.60	19 (1.9)	1.34 (0.49 to 3.64)	.56	48 (4.8)	0.76 (0.41 to 1.41)	.39
*P*_trend_§			.84			.59			.66			.41

* Adjusted for maternal age and parity. CNS = central nervous system; H = heritable; ICD = *International Classification of Diseases*, version 10; NH = nonheritable; RR = relative risk.

† Excludes preexisting diabetes mellitus (ICD 10: O24.0-O24.3) and unspecified whether preexisting or gestational diabetes (ICD 10: O24.9).

‡ Anemia complicating pregnancy, childbirth, and the puerperium.

§ *P* value based on two-sided Wald test.

‖ Relates to all female survivors not treated with any radiotherapy.

Survivors treated with abdominal radiotherapy were at 3.35-fold (95% CI = 1.41 to 7.93) risk of gestational diabetes mellitus compared with survivors treated without radiotherapy (Table 2), and this relative risk was increased for both Wilms tumor (RR = 2.73, 95% CI = 1.00 to 7.62) and non-Wilms tumor (RR = 4.27, 95% CI = 1.54 to 11.83) survivors. Overall, 4.9% of all survivors treated with abdominal radiotherapy developed diabetes mellitus during pregnancy, whereas this was only 1.5% in all survivors not treated with radiotherapy and 1.6% in the general population.

The relative risk of having developed anemia that complicates pregnancy was statistically significantly elevated among survivors treated with abdominal radiotherapy compared with survivors treated without radiotherapy (RR = 2.10, 95% CI = 1.27 to 3.46) and elevated for both Wilms (RR = 2.00, 95% CI = 1.13 to 3.57) and non-Wilms tumor (RR = 2.25, 95% CI = 1.13 to 4.49) survivors (Table 2). Eight point three percent of all survivors treated with abdominal radiotherapy experienced a pregnancy that was complicated by anemia, compared with 4.0% of survivors treated without radiotherapy and 4.4% in the general population.

### Maternal Care for Known or Suspected Disorder

Prior treatment with radiotherapy did not affect the likelihood of receiving specific maternal care for a known or suspected malpresentation of the fetus (*P*_heterogeneity_ = .96), a uterine scar from previous surgery (*P*_heterogeneity_ = .14), fetal problems such as poor fetal growth (*P*_heterogeneity_ = .66), or a prolonged pregnancy (*P*_heterogeneity_ = .58) (Table 3). Previous treatment with radiotherapy to the brain was associated with a small increased risk of premature rupture of the membranes (RR = 1.49, 95% CI = 1.07 to 2.08).

**Table 3.** Relative risk of receiving maternal care for problems relating to the fetus, amniotic cavity, and possible delivery problems among female survivors of childhood cancer

Characteristic	Maternal care related to:														
	Malpresentation of fetus* ICD10: O32			Uterine scar from previous surgery*,† ICD10: O34.2			Fetal problems*,‡ ICD10: O36			Premature rupture of membranes* ICD10: O42			Prolonged (post-term) pregnancy*,§,‖ ICD10: O48		
	No. (%)	RR (95% CI)	*P*¶	No. (%)	RR (95%CI)	*P*¶	No. (%)	RR (95% CI)	*P*¶	No. (%)	RR (95% CI)	*P*¶	No. (%)	RR (95% CI)	*P*¶
General population	1058 (4.2)	1.00 (ref.)		1699 (6.8)	1.00 (ref.)		1431 (5.7)	1.00 (ref.)		1913 (7.7)	1.00 (ref.)		1349 (5.4)	1.00 (ref.)	
Female survivors*#*	41 (4.8)	1.08 (0.78 to 1.62)	.42	76 (8.8)	1.23 (0.98 to 1.56)	.09	53 (6.2)	1.08 (0.82 to 1.41)	.65	54 (6.3)	0.83 (0.63 to 1.09)	.15	50 (5.8)	1.12 (0.82 to 1.49)	.50
Type of childhood cancer															
Leukemia	47 (5.1)	1.00 (ref.)		86 (9.4)	1.00 (ref.)		67 (7.3)	1.00 (ref.)		81 (8.9)	1.00 (ref.)		66 (7.2)	1.00 (ref.)	
Hodgkin lymphoma	9 (5.9)	1.10 (0.50 to 2.45)	.81	11 (7.2)	0.53 (0.23 to 1.20)	.13	11 (7.2)	1.00 (0.50 to 2.01)	.99	17 (11.1)	1.17 (0.73 to 1.89)	.51	7 (4.6)	0.59 (0.26 to 1.36)	.24
Non-Hodgkin lymphoma	4 (3.6)	0.66 (0.25 to 1.76)	.41	13 (11.7)	1.06 (0.59 to 1.92)	.84	7 (6.3)	0.87 (0.38 to 2.01)	.75	10 (9.0)	0.98 (0.51 to 1.88)	.94	6 (5.4)	0.74 (0.34 to 1.64)	.44
CNS tumor	19 (5.7)	1.04 (0.60 to 1.78)	.89	34 (10.1)	1.10 (0.75 to 1.62)	.62	20 (6.0)	0.81 (0.48 to 1.37)	.43	24 (7.1)	0.75 (0.49 to 1.15)	.19	22 (6.5)	0.84 (0.54 to 1.44)	.48
Neuroblastoma	4 (3.0)	0.62 (0.22 to 1.69)	.35	11 (8.1)	0.98 (0.51 to 1.86)	.95	15 (11.1)	1.48 (0.84 to 2.61)	.18	12 (8.9)	1.04 (0.57 to 1.90)	.91	2 (1.5)	0.16 (0.06 to 0.90)	.04
NH-retinoblastoma	4 (2.8)	0.50 (0.18 to 1.35)	.17	10 (7.0)	0.80 (0.43 to 1.46)	.46	6 (4.2)	0.59 (0.26 to 1.33)	.21	10 (7.0)	0.76 (0.41 to 1.43)	.40	12 (8.4)	1.13 (0.61 to 2.12)	.70
H-retinoblastoma	4 (6.9)	1.43 (0.54 to 3.81)	.47	2 (3.4)	0.36 (0.05 to 2.51)	.30	2 (3.4)	0.42 (0.11 to 1.60)	.20	3 (5.2)	0.56 (0.19 to 1.64)	.29	5 (8.6)	1.23 (0.53 to 2.84)	.65
Wilms tumor	18 (5.6)	1.11 (0.65 to 1.89)	.70	29 (9.0)	0.97 (0.63 to 1.50)	.89	30 (9.3)	1.35 (0.86 to 2.10)	.19	23 (7.1)	0.78 (0.50 to 1.23)	.29	11 (3.4)	0.52 (0.28 to 0.94)	.02
Bone tumor	6 (4.1)	0.81 (0.35 to 1.86)	.62	12 (8.3)	0.96 (0.54 to 1.69)	.89	5 (3.4)	0.49 (0.20 to 1.20)	.12	12 (8.3)	0.90 (0.49 to 1.65)	.72	8 (5.5)	0.74 (0.34 to 1.64)	.45
Soft-tissue sarcoma	9 (4.9)	0.94 (0.44 to 2.01)	.87	17 (9.2)	0.86 (0.51 to 1.45)	.57	13 (7.0)	0.95 (0.54 to 1.68)	.87	12 (6.5)	0.70 (0.37 to 1.33)	.27	15 (8.1)	1.04 (0.60 to 1.94)	.88
Other	13 (4.6)	0.88 (0.46 to 1.66)	.69	27 (9.6)	0.92 (0.58 to 1.44)	.71	12 (4.3)	0.61 (0.33 to 1.13)	.12	18 (6.4)	0.70 (0.42 to 1.16)	.16	11 (3.9)	0.48 (0.25 to 0.98)	.04
*P*_heterogeneity_¶			.89			.90			.15			.72			.12
Treated with radiotherapy															
No	41 (4.8)	1.00 (ref.)		76 (8.8)	1.00 (ref.)		53 (6.2)	1.00 (ref.)		54 (6.3)	1.00 (ref.)		50 (5.8)	1.00 (ref.)	
Brain	48 (5.3)	1.12 (0.73 to 1.72)	.60	86 (9.5)	1.15 (0.84 to 1.57)	.38	70 (7.8)	1.24 (0.86 to 1.79)	.25	82 (9.1)	1.49 (1.07 to 2.08)	.02	64 (7.1)	1.19 (0.82 to 1.76)	.30
Other (nonbrain/abdominal)	12 (5.2)	1.05 (0.52 to 2.10)	.90	16 (6.9)	0.66 (0.36 to 1.18)	.16	16 (6.9)	1.13 (0.65 to 1.97)	.67	19 (8.2)	1.27 (0.77 to 2.09)	.35	14 (6.1)	0.85 (0.46 to 1.75)	.77
Abdominal	17 (5.2)	1.07 (0.62 to 1.85)	.82	38 (11.7)	1.31 (0.88 to 1.93)	.18	24 (7.4)	1.26 (0.78 to 2.03)	.35	21 (6.4)	1.01 (0.61 to 1.68)	.96	18 (5.5)	0.94 (0.54 to 1.64)	.79
Abdominal non-Wilms	4 (3.4)	0.66 (0.24 to 1.81)	.42	15 (12.6)	1.41 (0.83 to 2.40)	.21	6 (5.0)	0.80 (0.32 to 2.04)	.65	9 (7.6)	1.18 (0.56 to 2.45)	.67	8 (6.7)	1.06 (0.54 to 2.29)	.86
Abdominal Wilms only	13 (6.3)	1.33 (0.72 to 2.43)	.36	23 (11.1)	1.26 (0.79 to 2.02)	.33	18 (8.7)	1.47 (0.88 to 2.47)	.14	12 (5.8)	0.93 (0.51 to 1.70)	.83	10 (4.8)	0.84 (0.44 to 1.64)	.58
No radiotherapy Wilms only	5 (5.2)	1.15 (0.46 to 2.91)	.76	6 (6.3)	0.82 (0.32 to 2.14)	.69	9 (9.4)	1.68 (0.75 to 3.79)	.21	8 (8.3)	1.47 (0.69 to 3.15)	.32	1 (1.0)	0.19 (0.03 to 1.36)	.10
*P*_heterogeneity_**¶**			.96			.14			.66			.09			.58
Age at diagnosis, y															
0–4	65 (4.9)	1.00 (ref.)		114 (8.7)	1.00 (ref.)		100 (7.6)	1.00 (ref.)		100 (7.6)	1.00 (ref.)		71 (6.3)	1.00 (ref.)	
5–9	37 (4.7)	0.88 (0.54 to 1.43)	.61	69 (8.8)	0.89 (0.62 to 1.27)	.51	56 (7.2)	1.07 (0.72 to 1.60)	.73	71 (9.1)	1.29 (0.91 to 1.83)	.15	48 (7.0)	1.24 (0.78 to 1.97)	.36
10–14	35 (5.1)	0.72 (0.39 to 1.33)	.30	69 (10.0)	0.92 (0.62 to 1.37)	.67	32 (4.7)	0.78 (0.47 to 1.30)	.35	51 (7.4)	0.92 (0.58 to 1.46)	.74	41 (7.0)	1.32 (0.77 to 2.27)	.32
*P*_trend_**¶**			.30			.63			.46			.99			.29
Decade of diagnosis															
<1980	49 (5.8)	1.00 (ref.)		112 (13.2)	1.00 (ref.)		58 (6.8)	1.00 (ref.)		58 (6.8)	1.00 (ref.)		46 (6.7)	1.00 (ref.)	
1980–1984	39 (4.8)	0.93 (0.56 to 1.55)	.78	74 (9.1)	0.72 (0.51 to 1.02)	.07	54 (6.7)	1.07 (0.67 to 1.73)	.77	65 (8.0)	1.23 (0.82 to 1.84)	.32	59 (8.4)	1.34 (0.82 to 2.20)	.24
1985–1991	41 (4.1)	0.87 (0.46 to 1.64)	.66	57 (5.8)	0.52 (0.33 to 0.81)	<.001	67 (6.8)	1.10 (0.65 to 1.88)	.72	91 (9.2)	1.35 (0.85 to 2.16)	.21	53 (6.0)	0.97 (0.53 to 1.80)	.93
*P*_trend_**¶**			.66			.004			.72			.22			.76

* Adjusted for maternal age and parity. CNS = central nervous system; H = heritable; ICD = *International Classification of Diseases*, version 10; NH = nonheritable; RR = relative risk.

† Includes maternal care due to scar previous cesarean section.

‡ Includes maternal care for rhesus isoimmunization, maternal care for poor fetal growth, maternal care for excessive fetal growth.

§ Deliveries via elective cesarean section excluded.

‖ Pregnancies that progressed beyond 42 weeks of gestation.

¶ *P* values were calculated using a two-sided Wald’s test.

# Relates to all female survivors not treated with any radiotherapy.

### Complications of Labor and Delivery

No statistically significant associations could be detected between any of the factors under study and the labor complications: abnormalities of forces of labor, long labor, obstructed labor, umbilical cord complications, delivery complicated by fetal stress (except for Wilms tumor survivors having a reduced risk of a delivery being complicated by fetal stress (RR = 0.73, 95% CI = 0.57 to 0.94) (Table 4)), perineal laceration, or postpartum hemorrhage (Table 5).

**Table 4.** Relative risk of developing specific complications of labor and delivery among female survivors of childhood cancer

Characteristic	Complications of labor and delivery														
	Abnormalities of forces of labor*,†,‡ ICD10: O62			Long labor‡,§,‖ ICD10: O63			Obstructed labor*,‡ ICD10: O64-O66			Delivery complicated by fetal stress*,¶ ICD10: O68			Umbilical cord complications* ICD10: O69		
	No. (%)	RR (95% CI)	*P*#	No. (%)	RR (95% CI)	*P*#	No. (%)	RR (95% CI)	*P*#	No. (%)	RR (95% CI)	*P*#	No. (%)	RR (95% CI)	*P*#
General population	720 (3.2)	1.00 (ref.)		2730 (12.0)	1.00 (ref.)		1007 (4.4)	1.00 (ref.)		5370 (23.7)	1.00 (ref.)		621 (2.7)	1.00 (ref.)	
Female survivors**	25 (3.3)	1.14 (0.74 to 1.58)	.75	104 (13.8)	1.21 (0.98 to 1.44)	.09	34 (4.5)	1.04 (0.72 to 1.54)	.83	191 (25.4)	1.10 (0.96 to 1.24)	.14	27 (3.6)	1.28 (0.86 to 1.88)	.15
Type of childhood cancer															
Leukemia	21 (2.6)	1.00 (ref.)		110 (13.8)	1.00 (ref.)		47 (5.9)	1.00 (ref.)		227 (28.4)	1.00 (ref.)		29 (3.6)	1.00 (ref.)	
Hodgkin lymphoma	10 (7.2)	2.25 (0.98 to 5.16)	.06	26 (18.7)	1.22 (0.83 to 1.79)	.32	7 (5.0)	0.85 (0.39 to 1.84)	.67	31 (22.3)	0.73 (0.52 to 1.02)	.06	6 (4.3)	1.21 (0.52 to 2.83)	.66
Non-Hodgkin lymphoma	1 (1.1)	0.36 (0.05 to 2.63)	.32	12 (12.8)	0.85 (0.48 to 1.52)	.59	3 (3.2)	0.53 (0.17 to 1.67)	.28	29 (30.9)	1.02 (0.73 to 1.42)	.92	2 (2.1)	0.60 (0.14 to 2.52)	.49
CNS tumor	16 (5.7)	1.93 (0.99 to 3.77)	.05	40 (14.3)	0.93 (0.67 to 1.30)	.68	16 (5.7)	0.95 (0.54 to 1.65)	.85	67 (24.0)	0.78 (0.62 to 1.00)	.05	12 (4.3)	1.20 (0.62 to 2.34)	.59
Neuroblastoma	3 (2.7)	1.06 (0.32 to 3.50)	.92	17 (15.2)	1.06 (0.67 to 1.67)	.80	1 (0.9)	0.15 (0.02 to 1.04)	.05	29 (25.9)	0.92 (0.66 to 1.29)	.64	1 (0.9)	0.25 (0.03 to 1.84)	.17
NH-retinoblastoma	6 (4.6)	1.60 (0.66 to 3.92)	.30	15 (11.5)	0.76 (0.44 to 1.31)	.32	8 (6.1)	1.06 (0.51 to 2.18)	.87	34 (26.0)	0.86 (0.61 to 1.21)	.39	7 (5.3)	1.50 (0.67 to 3.36)	.33
H-retinoblastoma	1 (1.9)	0.67 (0.09 to 4.88)	.69	3 (5.8)	0.43 (0.14 to 1.29)	.13	0 (0.0)	—	—	8 (15.4)	0.56 (0.30 to 1.03)	.06	1 (1.9)	0.53 (0.08 to 3.80)	.53
Wilms tumor	6 (2.2)	0.83 (0.34 to 2.05)	.69	29 (10.9)	0.76 (0.51 to 1.14)	.18	11 (4.1)	0.71 (0.37 to 1.35)	.29	55 (20.6)	0.73 (0.57 to 0.94)	.01	11 (4.1)	1.14 (0.58 to 2.25)	.70
Bone tumor	0 (0.0)	—	—	22 (19.0)	1.29 (0.87 to 1.92)	.21	6 (5.2)	0.85 (0.37 to 1.95)	.71	36 (31.0)	1.09 (0.82 to 1.45)	.53	5 (4.3)	1.19 (0.48 to 2.97)	.70
Soft-tissue sarcoma	3 (1.9)	0.66 (0.20 to 2.16)	.49	19 (11.9)	0.81 (0.50 to 1.30)	.37	12 (7.5)	1.30 (0.71 to 2.39)	.39	46 (28.9)	0.95 (0.72 to 1.26)	.71	3 (1.9)	0.53 (0.16 to 1.69)	.28
Other	9 (3.7)	1.28 (0.59 to 2.74)	.53	43 (17.6)	1.23 (0.89 to 1.69)	.21	9 (3.7)	0.63 (0.29 to 1.34)	.23	57 (23.3)	0.81 (0.63 to 1.04)	.09	6 (2.4)	0.68 (0.29 to 1.60)	.38
*P*_heterogeneity_**#**			.28			.24			.47			.09			.69
Treated with radiotherapy															
No	25 (3.3)	1.00 (ref.)		104 (13.8)	1.00 (ref.)		34 (4.5)	1.00 (ref.)		191 (25.4)	1.00 (ref.)		27 (3.6)	1.00 (ref.)	
Brain	20 (2.6)	0.86 (0.48 to 1.55)	.61	100 (13.0)	1.01 (0.78 to 1.31)	.94	45 (5.8)	1.30 (0.83 to 2.03)	.26	212 (27.5)	1.11 (0.94 to 1.31)	.24	28 (3.6)	0.99 (0.59 to 1.67)	.98
Other (nonbrain/abdominal)	8 (4.0)	1.04 (0.45 to 2.41)	.93	33 (16.5)	1.08 (0.74 to 1.57)	.69	8 (4.0)	0.86 (0.41 to 1.83)	.70	53 (26.5)	0.97 (0.74 to 1.27)	.82	11 (5.5)	1.56 (0.79 to 3.10)	.20
Abdominal	9 (3.5)	0.98 (0.44 to 2.17)	.96	33 (12.7)	0.93 (0.64 to 1.33)	.68	10 (3.8)	0.85 (0.43 to 1.69)	.64	56 (21.5)	0.84 (0.65 to 1.09)	.19	8 (3.1)	0.86 (0.40 to 1.86)	.71
Abdominal non-Wilms	5 (5.2)	1.37 (0.47 to 4.00)	.56	19 (19.8)	1.35 (0.87 to 2.09)	.18	6 (6.3)	1.38 (0.60 to 3.19)	.44	27 (28.1)	1.01 (0.71 to 1.43)	.96	2 (2.1)	0.56 (0.14 to 2.30)	.42
Abdominal Wilms only	4 (2.4)	0.74 (0.26 to 2.10)	.58	14 (8.5)	0.66 (0.39 to 1.11)	.12	4 (2.4)	0.54 (0.19 to 1.48)	.23	29 (17.7)	0.72 (0.51 to 1.03)	.07	6 (3.7)	1.01 (0.43 to 2.39)	.98
No radiotherapy Wilms only	2 (2.4)	0.81 (0.20 to 3.23)	.76	10 (11.9)	0.88 (0.44 to 1.77)	.73	3 (3.6)	0.76 (0.23 to 2.51)	.65	17 (20.2)	0.90 (0.59 to 1.37)	.61	4 (4.8)	1.46 (0.51 to 4.15)	.48
*P*_heterogeneity_**#**			.96			.93			.46			.18			.51
Age at diagnosis, y															
0–4	27 (2.4)	1.00 (ref.)		146 (13.0)	1.00 (ref.)		47 (4.2)	1.00 (ref.)		293 (26.0)	1.00 (ref.)		36 (3.2)	1.00 (ref.)	
5–9	24 (3.5)	1.29 (0.67 to 2.50)	.45	93 (13.6)	0.94 (0.71 to 1.25)	.67	36 (5.3)	1.08 (0.65 to 1.79)	.76	167 (24.4)	0.89 (0.73 to 1.09)	.27	24 (3.5)	0.90 (0.49 to 1.62)	.72
10–14	25 (4.3)	1.77 (0.82 to 3.85)	.15	96 (16.5)	0.90 (0.64 to 1.27)	.56	36 (6.2)	1.78 (1.05 to 3.04)	.03	159 (27.3)	1.02 (0.80 to 1.28)	.89	23 (4.0)	1.21 (0.62 to 2.34)	.57
*P*_trend_**#**			.15			.55			.05			.96			.63
Decade of diagnosis															
<1980	26 (3.8)	1.00 (ref.)		108 (15.7)	1.00 (ref.)		29 (4.2)	1.00 (ref.)		190 (27.7)	1.00 (ref.)		31 (4.5)	1.00 (ref.)	
1980–1984	20 (2.8)	1.12 (0.56 to 2.26)	.74	89 (12.6)	0.77 (0.57 to 1.03)	.08	35 (5.0)	1.27 (0.72 to 2.23)	.41	181 (25.6)	0.97 (0.78 to 1.21)	.80	26 (3.7)	1.21 (0.62 to 2.36)	.58
1985–1991	27 (3.1)	1.72 (0.79 to 3.76)	.17	119 (13.5)	0.76 (0.53 to 1.08)	.12	44 (5.0)	1.26 (0.66 to 2.41)	.49	215 (24.3)	0.87 (0.67 to 1.14)	.32	22 (2.5)	1.21 (0.53 to 2.76)	.66
*P*_trend_**#**			.16			.15			.54			.29			.66

* Adjusted for maternal age and parity. CNS = central nervous system; H = heritable; ICD = *International Classification of Diseases*, version 10; NH = nonheritable; RR = relative risk.

† For example, primary inadequate contractions; secondary uterine inertia; precipitate labor; hypertonic, incoordinate, and prolonged uterine contractions.

‡ Deliveries via elective cesarean section excluded.

§ Long labor includes: prolonged first stage (ICD10: O63.0), prolonged second stage (ICD10: O63.1), and prolonged labor not otherwise specified (ICD10: O63.9).

‖ Adjusted for: maternal age, parity, birthweight, and gestational age.

¶ For example, labor and delivery complicated by fetal heart rate anomaly, meconium in amniotic fluid, or other evidence of fetal stress.

# *P* values were calculated using a two-sided Wald test.

** Relates to all female survivors not treated with any radiotherapy

**Table 5.** Relative risk of developing specific complications of labor and delivery among female survivors of childhood cancer

Characteristic	Complications of labor and delivery						Delivery						Supervision of high-risk pregnancy* ICD10: Z35		
	Perineal laceration*,† ICD10: O70			Postpartum hemorrhage* ICD10: O72			Elective cesarean section*,†			Emergency cesarean section*,†					
	No. (%)	RR (95% CI)	*P*‡	No. (%)	RR (95% CI)	*P*‡	No. (%)	RR (95% CI)	*P*‡	No. (%)	RR (95% CI)	*P*‡	No. (%)	RR (95% CI)	*P*‡
General population	9229 (40.7)	1.00 (ref.)		2179 (8.7)	1.00 (ref.)		2249 (10.4)	1.00 (ref.)		3174 (14.0)	1.00 (ref.)		999 (4.0)	1.00 (ref.)	
Female survivors§	313 (41.7)	0.99 (0.89 to 1.10)	.75	82 (9.5)	1.08 (0.93 to 1.28)	.46	109 (14.7)	1.39 (1.16 to 1.70)	<.001	119 (15.8)	1.08 (0.91 to 1.27)	.21	41 (4.8)	1.19 (0.85 to 1.64)	.32
Type of childhood cancer															
Leukemia	307 (38.4)	1.00 (ref.)		93 (10.2)	1.00 (ref.)		116 (15.2)	1.00 (ref.)		148 (18.6)	1.00 (ref.)		34 (3.7)	1.00 (ref.)	
Hodgkin lymphoma	63 (45.3)	1.16 (0.94 to 1.43)	.17	18 (11.8)	1.03 (0.62 to 1.69)	.92	14 (10.3)	0.59 (0.32 to 1.08)	.09	17 (12.2)	0.59 (0.36 to 0.97)	.04	8 (5.2)	0.97 (0.44 to 2.18)	.95
Non-Hodgkin lymphoma	30 (31.9)	0.81 (0.59 to 1.11)	.19	10 (9.0)	0.79 (0.37 to 1.69)	.55	17 (18.7)	1.09 (0.64 to 1.84)	.75	20 (21.3)	1.03 (0.67 to 1.60)	.88	4 (3.6)	0.88 (0.33 to 2.39)	.80
CNS tumor	109 (39.1)	1.03 (0.86 to 1.24)	.79	33 (9.8)	0.87 (0.59 to 1.28)	.47	57 (20.1)	1.22 (0.90 to 1.65)	.20	51 (18.3)	0.90 (0.67 to 1.22)	.51	12 (3.6)	0.77 (0.40 to 1.47)	.43
Neuroblastoma	43 (38.4)	1.04 (0.80 to 1.34)	.80	17 (12.6)	1.27 (0.75 to 2.16)	.37	23 (18.7)	1.20 (0.74 to 1.96)	.46	12 (10.7)	0.64 (0.37 to 1.11)	.11	5 (3.7)	1.04 (0.39 to 2.72)	.94
NH-retinoblastoma	53 (40.5)	1.09 (0.86 to 1.39)	.50	14 (9.8)	0.89 (0.52 to 1.53)	.68	12 (9.8)	0.71 (0.41 to 1.22)	.21	21 (16.0)	0.78 (0.50 to 1.21)	.26	7 (4.9)	0.93 (0.41 to 2.12)	.86
H-retinoblastoma	26 (50.0)	1.28 (0.88 to 1.86)	.20	6 (10.3)	1.00 (0.46 to 2.17)	.99	6 (11.5)	0.77 (0.33 to 1.83)	.55	6 (11.5)	0.68 (0.31 to 1.46)	.32	1 (1.7)	0.37 (0.05 to 2.72)	.33
Wilms tumor	102 (38.2)	0.99 (0.81 to 1.21)	.91	41 (12.7)	1.23 (0.86 to 1.76)	.26	55 (20.0)	1.23 (0.88 to 1.73)	.22	47 (17.6)	0.97 (0.71 to 1.31)	.83	16 (5.0)	1.18 (0.62 to 2.26)	.61
Bone tumor	46 (39.7)	1.01 (0.78 to 1.30)	.96	10 (6.9)	0.63 (0.34 to 1.17)	.14	29 (22.5)	1.52 (1.05 to 2.20)	.03	16 (13.8)	0.77 (0.47 to 1.26)	.30	5 (3.4)	0.86 (0.35 to 2.13)	.75
Soft-tissue sarcoma	52 (32.7)	0.85 (0.65 to 1.11)	.21	18 (9.7)	0.87 (0.54 to 1.42)	.58	26 (17.2)	1.07 (0.70 to 1.63)	.76	33 (20.9)	1.04 (0.73 to 1.50)	.81	8 (4.3)	0.89 (0.40 to 1.99)	.78
Other	95 (38.8)	1.00 (0.82 to 1.21)	.93	21 (7.5)	0.69 (0.45 to 1.08)	.10	35 (14.7)	0.87 (0.58 to 1.29)	.48	42 (17.1)	0.89 (0.64 to 1.24)	.48	14 (5.0)	1.12 (0.60 to 2.09)	.72
*P*_heterogeneity_‡			.59			.46			.10			.50			.99
Treated with radiotherapy															
No	313 (41.7)	1.00 (ref.)		82 (9.5)	1.00 (ref.)		109 (14.7)	1.00 (ref.)		119 (15.8)	1.00 (ref.)		41 (4.8)	1.00 (ref.)	
Brain	296 (38.3)	0.91 (0.80 to 1.05)	.21	93 (10.3)	1.14 (0.85 to 1.53)	.37	129 (17.0)	1.15 (0.90 to 1.49)	.27	138 (17.9)	1.21 (0.95 to 1.53)	.12	32 (3.6)	0.88 (0.55 to 1.40)	.58
Other (nonbrain/abdominal)	78 (39.0)	0.94 (0.76 to 1.15)	.55	23 (10.0)	0.98 (0.62 to 1.55)	.93	31 (15.7)	0.90 (0.59 to 1.37)	.63	33 (16.5)	0.97 (0.67 to 1.40)	.87	12 (5.2)	0.95 (0.49 to 1.84)	.88
Abdominal	97 (37.3)	0.88 (0.72 to 1.07)	.20	41 (12.6)	1.33 (0.93 to 1.89)	.12	66 (24.4)	1.46 (1.07 to 1.99)	.02	56 (21.5)	1.35 (1.00 to 1.83)	.05	17 (5.2)	1.04 (0.58 to 1.87)	.90
Abdominal non-Wilms	35 (36.5)	0.83 (0.61 to 1.13)	.23	15 (12.6)	1.25 (0.75 to 2.07)	.39	23 (24.0)	1.36 (0.87 to 2.13)	.18	23 (24.0)	1.36 (0.87 to 2.11)	.17	6 (5.0)	0.82 (0.33 to 2.07)	.68
Abdominal Wilms only	62 (37.8)	0.90 (0.70 to 1.14)	.37	26 (12.6)	1.37 (0.89 to 2.10)	.15	43 (24.7)	1.46 (1.01 to 2.11)	.05	33 (20.1)	1.32 (0.92 to 1.89)	.13	11 (5.3)	1.16 (0.58 to 2.33)	.68
No radiotherapy Wilms only	34 (40.5)	0.98 (0.71 to 1.35)	.90	11 (11.5)	1.37 (0.74 to 2.53)	.31	12 (14.1)	1.10 (0.57 to 2.12)	.78	11 (13.1)	1.06 (0.59 to 1.90)	.85	5 (5.2)	1.39 (0.43 to 4.50)	.58
*P*_heterogeneity_‡			.49			.42			.07			.14			.94
Age at diagnosis, y															
0–4	427 (37.9)	1.00 (ref.)		132 (10.0)	1.00 (ref.)		189 (16.8)	1.00 (ref.)			1.00 (ref.)		51 (3.9)	1.00 (ref.)	
5–9	277 (40.4)	1.01 (0.86 to 1.18)	.93	83 (10.6)	1.13 (0.81 to 1.58)	.47	96 (14.4)	0.79 (0.60 to 1.05)	.11	111 (16.3)	0.92 (0.70 to 1.21)	.57	21 (2.7)	0.69 (0.38 to 1.25)	.22
10–14	220 (37.8)	0.96 (0.80 to 1.16)	.69	66 (9.6)	1.08 (0.71 to 1.63)	.73	105 (18.3)	0.85 (0.61 to 1.19)	.35	113 (19.4)	1.15 (0.84 to 1.56)	.36	42 (6.1)	1.30 (0.78 to 2.17)	.31
*P*_trend_‡			.73			.65			.26			.46			.35
Decade of diagnosis															
<1980	276 (40.2)	1.00 (ref.)		91 (10.7)	1.00 (ref.)		162 (22.3)	1.00 (ref.)		120 (17.5)	1.00 (ref.)		45 (5.3)	1.00 (ref.)	
1980–1984	277 (39.2)	1.03 (0.87 to 1.23)	.72	82 (10.1)	0.89 (0.63 to 1.27)	.52	103 (15.1)	0.70 (0.52 to 0.94)	.02	127 (18.0)	1.12 (0.84 to 1.50)	.43	38 (4.7)	1.35 (0.78 to 2.32)	.28
1985–1991	333 (37.7)	1.06 (0.87 to 1.31)	.55	97 (9.8)	0.88 (0.59 to 1.34)	.56	108 (12.7)	0.58 (0.40 to 0.83)	<.001	140 (15.9)	0.92 (0.64 to 1.31)	.64	30 (3.0)	1.21 (0.65 to 2.26)	.54
*P*_trend_‡			.54			.59			.004			.52			.52

* Adjusted for maternal age and parity. CNS = central nervous system; H = heritable; ICD = *International Classification of Diseases*, version 10; NH = nonheritable; RR = relative risk.

† Compared with vaginal delivery.

‡ *P* value based on two-sided Wald test.

§ Relates to all female survivors not treated with any radiotherapy.

### Cesarean Delivery and Supervision High-Risk Pregnancy

Compared with the general population, survivors treated without radiotherapy were 39% more likely to opt for an elective cesarean (RR = 1.39, 95% CI = 1.16 to 1.70). Particularly survivors of a bone tumor (RR = 1.52, 95% CI = 1.05 to 2.20) and those treated with abdominal radiotherapy (RR = 1.46, 95% CI = 1.07 to 1.99) (Table 5) were more likely to opt for a cesarean. Survivors treated longer ago were more likely to undergo an elective cesarean than more recently treated survivors (22.3% before 1980 vs 12.7% in 1985 to 1991, *P*_trend_ = .004). The risk of an emergency cesarean was not elevated among survivors compared with the general population (*P* = .21), although survivors of Hodgkin lymphoma appeared to be at reduced risk (RR = 0.59, 95% CI = 0.36 to 0.97). Pregnancies in survivors treated with abdominal radiotherapy were not identified as high-risk pregnancies requiring greater supervision than pregnancies in survivors treated without radiotherapy (RR = 1.04, 95% CI = 0.58 to 1.87).

### Adverse Pregnancy Outcomes

Wilms tumor survivors treated with abdominal radiotherapy were at threefold risk of delivering offspring with a low birthweight compared with survivors treated without radiotherapy (RR = 2.85, 95% CI = 1.79 to 4.48) (Supplementary Table 1, available online). The RR for preterm delivery was also statistically significantly increased for Wilms tumor survivors (RR = 1.89, 95% CI = 1.30 to 2.74). Only 19 hospital admissions were related to a stillbirth, and no stillbirths were recorded among women treated with abdominal radiotherapy.

### Sensitivity Analysis by HES Calendar Year

No statistically significant variation was found in the relative risk of developing a pregnancy or labor complication by HES calendar year (Supplementary Table 2, available online), suggesting that if there was potential underreporting of any pregnancy or labor outcomes before HES year 2002, the effect would have been minimal.

## Discussion

To our knowledge, this is the largest study investigating the risks of pregnancy and labor complications in childhood cancer survivors. This study shows that treatment with abdominal radiotherapy increases the risk of developing hypertension complicating pregnancy in Wilms tumor survivors, as well as diabetes mellitus and anemia complicating pregnancy in all survivors. In addition, female survivors as a whole are more likely to opt for an elective cesarean than the general population. Our results of increased risks of preterm delivery and babies born with low birthweight among female survivors of childhood cancer treated with abdominal radiotherapy concur with previously reported data (4–10).

In this study, hypertensive disorders complicated 23.7% of all pregnancies among Wilms tumor survivors treated with abdomino-pelvic radiotherapy. Consistent with these findings, Green et al. (13) reported that 23.7% of all pregnancies among Wilms tumor survivors treated with irradiation were complicated by hypertension. There is a possibility that the risk of hypertension complicating pregnancy may be related to a previous nephrectomy rather than actual abdominal radiotherapy; however, because the vast majority of Wilms tumor survivors would have undergone a nephrectomy (19,20), the risks should then, in theory, also be increased among those Wilms tumor survivors not treated with radiotherapy. However, this was not supported by our data—only 9.4% of Wilms tumor survivors not treated with abdominal radiotherapy developed hypertension complicating pregnancy; not statistically significantly different than that observed in the general population (6.0%) or than other survivors not treated with radiotherapy (7.1%).

The exact biological mechanism explaining the risk of hypertension and anemia complicating pregnancy after abdominal radiotherapy is poorly understood. Hypertension, anemia, and varying degrees of chronic glomerular impairment are well documented features of chronic radiation-induced renal injury, which may also reduce a survivor’s reserve against future renal stresses. Statistically significant glomerular impairment has been reported in 25% to 56% of children receiving renal doses of 12 to 24 Gy (21). It is plausible that hypertension and anemia may be recognized for the first time during pregnancy in some female survivors with less severe degrees of chronic radiation-induced renal damage as a result of the greater physiological stresses and increased medical surveillance during pregnancy.

To our knowledge, this is the first study to report an elevated risk of developing diabetes in pregnancy among survivors treated with abdominal radiotherapy. A linked cancer-birth registry analysis from four US centers (8) showed in an exploratory analysis that female bone cancer survivors were at risk of diabetes during pregnancy (RR = 4.92, 95% CI = 1.60 to 15.13), but this was based on small observed numbers. Other studies have found that survivors of childhood cancer treated with abdominal radiation are at risk of developing diabetes (22–25), but not specifically during pregnancy. The mechanism for developing diabetes mellitus is unclear but may relate to a radiation-induced effect on the pancreas, perhaps causing inflammation and fibrosis, which may reduce subsequent insulin secretion from the islet cells.

In this study, survivors were more likely to opt for an elective cesarean than the general population. In a recent smaller study, Melin et al. (26) observed a 50% increased odds among 456 survivors of childhood cancer for undergoing a cesarean delivery compared with siblings which is consistent with our findings. It is not clear why survivors are more likely to opt for an elective cesarean, but it could be indicative of the obstetrician aiming to reduce any theoretical risk that a vaginal delivery might have. For example, survivors previously exposed to treatment modalities known to be associated with cardiomyopathy (eg, chest irradiation and high-dose cumulative doses of anthracycline) (27) might have opted for a cesarean delivery to decrease the potential risk relating to cardiomyopathy during labor and puerperium.

Several potential limitations should be considered. First, large-scale linkage exercises may suffer from inaccuracies with regard to linking the correct patient to the corresponding health records. However, because linkage of the BCCSS cohort with HES was done using NHS number, date of birth, and postcode of each patient, such inaccuracies in linkage are likely to be minimal. Lack of detailed treatment exposure, such as administered chemotherapy and radiation treatment charts, did not allow for conducting detailed dose-response analyses. However, we did consider Wilms tumor survivors separately who, if treated with radiotherapy, would have received one of the highest doses of radiation to the abdomen of all childhood cancer survivors. Evaluation of potential confounding of the association between abdominal irradiation and specific pregnancy and labor complications by chemotherapy was also not possible. Investigation of pregnancy and labor complications by detailed treatment exposure would require a nested case-control study. Last, we acknowledge that information on site of radiotherapy was missing for 16.9% of all survivors.

A major strength of the current study is the population-based design, which overcomes a variety of potential limitations including the issue of selection bias related to ascertainment of survivors of childhood cancer into the cohort. Hospital-based studies are probably more likely to include those survivors who were treated more intensely, and any absolute risk estimates of adverse late effects—including pregnancy outcomes—are therefore more likely to be overestimated than within a population-based study. It also provided us with the opportunity to compare the risks of pregnancy and labor outcomes with the general population in an entirely population-based way. Additionally, record linkage of our cohort with the population-based HES provides ascertainment of pregnancy and labor outcomes in a systematic way, unlike studies that ascertain adverse outcomes through either (postal) questionnaires or hospital records, which may suffer from nonresponse or selection bias, respectively.

It has previously been shown that uterine damage, manifested by impaired growth and blood flow, is a likely consequence of abdomino-pelvic irradiation (12,28,29) and that uterine volume correlates with age at irradiation (30). Exposure of the pelvis to radiation is associated with risk of miscarriage, delivering prematurely, and low-birthweight offspring, and in this study we have shown further evidence that there is a risk of hypertension complicating pregnancy in Wilms tumor survivors, and diabetes mellitus and anemia complicating pregnancy in all survivors who have received abdominal radiotherapy. Although survivors treated with abdominal radiotherapy were more likely to opt for an elective cesarean, the risks of specific complications during labor were not statistically significantly increased, and there is thus no evidence base for suggesting that an elective cesarean should be the optimum mode of delivery in survivors of childhood cancer treated with abdominal radiotherapy.

In conclusion, the results of this study into pregnancy and labor complications among female survivors of childhood cancer show that treatment with abdominal radiotherapy increases the risk of developing hypertension complicating pregnancy in Wilms tumor survivors, and diabetes mellitus and anemia complicating pregnancy in all survivors. These patients may require extra vigilance during pregnancy.

## Funding

This work was supported by grant number C386/A10422 (MMH) and C386/A11709 (MMH/RCR) from Cancer Research UK and a grant from the European Union’s 7th Framework Programme for research, technological development, and demonstration under grant agreement no. 257505 (MMH).

## Notes

Neither funder had a role in the study design; collection, analysis, or interpretation of the data; the writing of the report; or the decision to submit the article for publication. Parts of this manuscript have previously been presented at the 14th International Conference on Long-Term Complications of Treatment of Children and Adolescents for Cancer, June 11–13, 2015, Arlington, Virginia.

The British Childhood Cancer Survivor Study (BCCSS) is a national collaborative undertaking guided by a Steering Group that comprises Douglas Easton (Chair), Michael Hawkins, Helen Jenkinson, Meriel Jenney, Raoul Reulen, Kathryn Pritchard-Jones, Elaine Sugden, Andrew Toogood, and Hamish Wallace. There is a survivor representative on the Steering Group, Ms. Alexandra Brownsdon. The BCCSS benefits from the contributions of the Officers, Centers, and individual members of the Children’s Cancer and Leukaemia Group and the Regional Pediatric Cancer Registries. The BCCSS acknowledges the collaboration of the Office for National Statistics, the National Records of Scotland, the Welsh Cancer Intelligence and Surveillance Unit, the Health and Social Care Information Centre, and Public Health England. The BCCSS would not have been possible without the financial support of Cancer Research UK and the European Commission, to which we offer profound thanks. The views expressed in this publication are those of the authors and do not necessarily represent those of the funders or collaborators. Finally, thanks to all BCCSS staff who have given many years of dedicated work to bring the BCCSS to fruition.

## Supplementary Material

Supplementary Data

## References

[1] Gatta G, Botta L, Rossi S, Childhood cancer survival in Europe 1999–2007: Results of EUROCARE-5—a population-based study. Lancet Oncol. 2014;15(1):35–47. 24314616 10.1016/S1470-2045(13)70548-5

[2] Oeffinger KC, Mertens AC, Sklar CA, Chronic health conditions in adult survivors of childhood cancer. N Engl J Med. 2006;355(15):1572–1582. 17035650 10.1056/NEJMsa060185

[3] Geenen MM, Cardous-Ubbink MC, Kremer LC, Medical assessment of adverse health outcomes in long-term survivors of childhood cancer. JAMA. 2007;297(24):2705–2715. 17595271 10.1001/jama.297.24.2705

[4] Chiarelli AM, Marrett LD, Darlington GA. Pregnancy outcomes in females after treatment for childhood cancer. Epidemiology. 2000;11(2):161–166. 11021613 10.1097/00001648-200003000-00013

[5] Green DM, Peabody EM, Nan B, Peterson S, Kalapurakal JA, Breslow NE. Pregnancy outcome after treatment for Wilms tumor: A report from the National Wilms Tumor Study Group. J Clin Oncol. 2002;20(10):2506–2513. 12011129 10.1200/JCO.2002.07.159

[6] Green DM, Whitton JA, Stovall M, Pregnancy outcome of female survivors of childhood cancer: A report from the Childhood Cancer Survivor Study. Am J Obstet Gynecol. 2002;187(4):1070–1080. 12389007 10.1067/mob.2002.126643

[7] Signorello LB, Cohen SS, Bosetti C, Female survivors of childhood cancer: Preterm birth and low birth weight among their children. J Natl Cancer Inst. 2006;98(20):1453–1461. 17047194 10.1093/jnci/djj394 PMC2730161

[8] Mueller BA, Chow EJ, Kamineni A, Pregnancy outcomes in female childhood and adolescent cancer survivors: A linked cancer-birth registry analysis. Arch Pediatr Adolesc Med. 2009;163(10):879–886. 19805705 10.1001/archpediatrics.2009.112 PMC2758647

[9] Reulen RC, Zeegers MP, Wallace WH, Pregnancy outcomes among adult survivors of childhood cancer in the British Childhood Cancer Survivor Study. Cancer Epidemiol Biomarkers Prev. 2009;18(8):2239–2247. 19661083 10.1158/1055-9965.EPI-09-0287 PMC2730581

[10] Madanat-Harjuoja LM, Malila N, Lahteenmaki PM, Boice JD Jr, Gissler M, Dyba T. Preterm delivery among female survivors of childhood, adolescent and young adulthood cancer. Int J Cancer. 2010;127(7):1669–1679. 20054856 10.1002/ijc.25157 PMC2919618

[11] Green DM, Fine WE, Li FP. Offspring of patients treated for unilateral Wilms' tumor in childhood. Cancer. 1982;49(11):2285–2288. 6280837 10.1002/1097-0142(19820601)49:11<2285::aid-cncr2820491114>3.0.co;2-n

[12] Larsen EC, Schmiegelow K, Rechnitzer C, Loft A, Muller J, Andersen AN. Radiotherapy at a young age reduces uterine volume of childhood cancer survivors. Acta Obstet Gynecol Scand. 2004;83(1):96–102. 14678092 10.1111/j.1600-0412.2004.00332.x

[13] Green DM, Lange JM, Peabody EM, Pregnancy outcome after treatment for Wilms tumor: A report from the national Wilms tumor long-term follow-up study. J Clin Oncol. 2010;28(17):2824–2830. 20458053 10.1200/JCO.2009.27.2922 PMC2903317

[14] Hawkins MM, Lancashire ER, Winter DL, The British Childhood Cancer Survivor Study: Objectives, methods, population structure, response rates and initial descriptive information. Pediatr Blood Cancer. 2008;50(5):1018–1025. 17849473 10.1002/pbc.21335

[15] Abrahams C, Davy K. Linking HES Maternity Records with ONS Birth Records. Health Stat Q. 2002;(13):22–30.

[16] Dattani N, Datta-Nemdharry P, Macfarlane A. Linking maternity data for England 2007: Methods and data quality. Health Stat Q. 2011;(49):53–79. 10.1057/hsq.2011.3 21372845

[17] Ghosh RE, Ashworth DC, Hansell AL, Garwood K, Elliott P, Toledano MB. Routinely collected English birth data sets: Comparisons and recommendations for reproductive epidemiology. Arch Dis Child Fetal Neonatal Ed. 2016;101(5):F451–F457. 26837309 10.1136/archdischild-2015-309540

[18] StataCorp. Stata Statistical Software: Release 14. College Station, TX: StataCorp LP 2015.

[19] Pritchard J, Imeson J, Barnes J, Results of the United Kingdom Children's Cancer Study Group first Wilms' Tumor Study. J Clin Oncol. 1995;13(1):124–133. 7799012 10.1200/JCO.1995.13.1.124

[20] Mitchell C, Jones PM, Kelsey A, The treatment of Wilms' tumour: Results of the United Kingdom Children's cancer study group (UKCCSG) second Wilms' tumour study. Br J Cancer. 2000;83(5):602–608. 10944599 10.1054/bjoc.2000.1338 PMC2363501

[21] Dawson LA, Kavanagh BD, Paulino AC, Radiation-associated kidney injury. Int J Radiat Oncol Biol Phys. 2010;76(3 suppl):S108–S115. 20171504 10.1016/j.ijrobp.2009.02.089

[22] van Nimwegen FA, Schaapveld M, Janus CP, Risk of diabetes mellitus in long-term survivors of Hodgkin lymphoma. J Clin Oncol. 2014;32(29):3257–3263. 25154821 10.1200/JCO.2013.54.4379

[23] Holmqvist AS, Olsen JH, Andersen KK, Adult life after childhood cancer in Scandinavia: Diabetes mellitus following treatment for cancer in childhood. Eur J Cancer. 2014;50(6):1169–1175. 24507548 10.1016/j.ejca.2014.01.014

[24] de Vathaire F, El-Fayech C, Ben Ayed FF, Radiation dose to the pancreas and risk of diabetes mellitus in childhood cancer survivors: A retrospective cohort study. Lancet Oncol. 2012;13(10):1002–1010. 22921663 10.1016/S1470-2045(12)70323-6

[25] Meacham LR, Sklar CA, Li S, Diabetes mellitus in long-term survivors of childhood cancer. Increased risk associated with radiation therapy: A report for the childhood cancer survivor study. Arch Intern Med. 2009;169(15):1381–1388. 19667301 10.1001/archinternmed.2009.209 PMC3529471

[26] Melin J, Heinavaara S, Malila N, Tiitinen A, Gissler M, Madanat-Harjuoja L. Adverse obstetric outcomes among early-onset cancer survivors in Finland. Obstet Gynecol. 2015;126(4):803–810. 26348187 10.1097/AOG.0000000000001035

[27] Adams MJ, Hardenbergh PH, Constine LS, Lipshultz SE. Radiation-associated cardiovascular disease. Crit Rev Oncol Hematol. 2003;45(1):55–75. 12482572 10.1016/s1040-8428(01)00227-x

[28] Bath LE, Critchley HO, Chambers SE, Anderson RA, Kelnar CJ, Wallace WH. Ovarian and uterine characteristics after total body irradiation in childhood and adolescence: Response to sex steroid replacement. Br J Obstet Gynaecol. 1999;106(12):1265–1272. 10609720 10.1111/j.1471-0528.1999.tb08180.x

[29] Critchley HO, Wallace WH, Shalet SM, Mamtora H, Higginson J, Anderson DC. Abdominal irradiation in childhood; the potential for pregnancy. Br J Obstet Gynaecol 1992;99(5):392–394. 1622911 10.1111/j.1471-0528.1992.tb13755.x

[30] Critchley HO, Bath LE, Wallace WH. Radiation damage to the uterus—review of the effects of treatment of childhood cancer. Hum Fertil (Camb). 2002;5(2):61–66. 12082209 10.1080/1464727022000198942

