# Supplementary material for: Pregnancy and Labor Complications in Female Survivors of Childhood Cancer: The British Childhood Cancer Survivor Study
Source: J Natl Cancer Inst. 2017 Apr 17;109(11):djx056. doi: 10.1093/jnci/djx056 (PMC5409032; doi:10.1093/jnci/djx056)
Supplement: Supplementary Data [file djx056_Supp.docx]

**SUPPLEMENTARY MATERIALS**

**Pregnancy and labour complications in female survivors of childhood cancer: The British Childhood Cancer Survivor Study**

**Authors:** Raoul C Reulen^1,*^, Chloe J Bright^1^, Dave L Winter^1^, Miranda M Fidler^1^, Kwok Wong^1^, Joyeeta Guha^1^, Julie S Kelly^1^, Clare Frobisher^1^, Angela B Edgar^2^, Roderick Skinner^3^, W Hamish B Wallace^2^, Mike M Hawkins^1^

^1^Centre for Childhood Cancer Survivor Studies, Institute of Applied Health Research, Robert Aitken Building, University of Birmingham, Edgbaston, Birmingham B15 2TT, UK

^2^Department of Paediatric Haematology and Oncology, Royal Hospital for Sick Children, University of Edinburgh, Edinburgh EH9 1LF

^3^Department of Paediatric and Adolescent Haematology and Oncology, and Children’s BMT Unit, Great North Children’s Hospital, Royal Victoria Infirmary, Newcastle upon Tyne, UK

^*^Correspondence to: Raoul C. Reulen, email: [r.c.reulen@bham.ac.uk](mailto:r.c.reulen@bham.ac.uk), Telephone: +441214144946, Centre for Childhood Cancer Survivor Studies, Institute of Applied Health Research, Robert Aitken Building, University of Birmingham, Edgbaston, Birmingham B15 2TT, UK

**Supplementary Table 1.** Relative risk (RR) of adverse pregnancy outcomes and number and percentage of still births.

| **Characteristics** | **Adverse pregnancy outcomes** | | | | | | **Still-birth**† |
| --- | --- | --- | --- | --- | --- | --- | --- |
|  | **Low birth weight*** | | | **Premature delivery**^*^ | | |  |
|  | **No. (%)** | **RR (95%CI)** | **P‡** | **No. (%)** | **RR (95%CI)** | **P‡** | **No. (%)** |
| General population | 1255 (6.5%) | 1.00 (ref.) |  | 1167 (7.0%) | 1.00 (ref.) |  | 148 (0.6%) |
| Female survivors ^§^ | 49 (7.7%) | 1.22 (0.85,1.63) | 0.26 | 81 (14.2%) | 2.15 (1.74,2.74) | <0.001 | 7 (0.8%) |
| Type of childhood cancer |  |  |  |  |  |  |  |
| Leukaemia | 65 (9.1%) | 1.00 (ref.) |  | 72 (11.6%) | 1.00 (ref.) |  | 6 (0.7%) |
| Hodgkin's lymphoma | 4 (3.6%) | 0.44 (0.16,1.20) | 0.11 | 5 (5.3%) | 0.43 (0.18,1.03) | 0.06 | 0 (0.0%) |
| Non-Hodgkin lymphoma | 7 (7.8%) | 0.93 (0.44,1.97) | 0.85 | 12 (15.6%) | 1.25 (0.65,2.40) | 0.50 | 1 (0.9%) |
| CNS tumour | 27 (10.9%) | 1.36 (0.84,2.21) | 0.21 | 38 (16.7%) | 1.48 (0.98,2.22) | 0.06 | 2 (0.6%) |
| Neuroblastoma | 15 (15.3%) | 1.68 (0.94,3.00) | 0.08 | 21 (21.2%) | 1.74 (1.07,2.84) | 0.03 | 4 (3.0%) |
| NH-retinoblastoma | 8 (6.9%) | 0.68 (0.29,1.60) | 0.38 | 8 (8.1%) | 0.69 (0.30,1.59) | 0.38 | 1 (0.7%) |
| H-retinoblastoma | 3 (7.9%) | 1.02 (0.33,3.11) | 0.98 | 4 (12.5%) | 1.21 (0.47,3.15) | 0.70 | 0 (0.0%) |
| Wilms tumour | 44 (17.4%) | 2.02 (1.34,3.05) | <0.001 | 54 (23.5%) | 2.10 (1.47,2.99) | <0.001 | 1 (0.3%) |
| Bone tumour | 7 (6.8%) | 0.79 (0.34,1.84) | 0.58 | 13 (13.1%) | 1.13 (0.62,2.07) | 0.69 | 0 (0.0%) |
| Soft-tissue sarcoma | 19 (13.8%) | 1.72 (1.00,2.95) | 0.05 | 24 (20.9%) | 1.82 (1.15,2.89) | 0.01 | 1 (0.5%) |
| Other | 19 (8.9%) | 1.01 (0.58,1.75) | 0.98 | 29 (14.9%) | 1.41 (0.93,2.15) | 0.11 | 3 (1.1%) |
| *P_heterogeneity_*‡ |  |  | 0.006 |  |  | 0.001 |  |
| Treated with radiotherapy |  |  |  |  |  |  |  |
| No | 49 (7.7%) | 1.00 (ref.) |  | 81 (14.2%) | 1.0 (ref.) |  | 7 (0.8%) |
| Brain | 69 (9.8%) | 1.28 (0.86,1.90) | 0.19 | 80 (13.1%) | 0.89 (0.65,1.22) | 0.48 | 6 (0.7%) |
| Other (non-brain/abdominal) | 25 (15.0%) | 2.05 (1.25,3.35) | <0.001 | 24 (15.8%) | 1.20 (0.77,1.85) | 0.42 | 2 (0.9%) |
| Abdominal | 42 (17.1%) | 2.31 (1.50,3.55) | <0.001 | 53 (24.0%) | 1.70 (1.21,2.38) | <0.001 | 0 (0.0%) |
| Abdominal non-wilms | 9 (10.2%) | 1.40 (0.67,2.90) | 0.38 | 16 (19.5%) | 1.34 (0.77,2.32) | 0.30 | 0 (0.0%) |
| Abdominal Wilms only | 33 (21.0%) | 2.85 (1.79,4.48) | <0.001 | 37 (26.6%) | 1.89 (1.30,2.74) | <0.001 | 0 (0.0%) |
| No radiotherapy Wilms only | 10 (12.8%) | 1.75 (0.85,3.65) | 0.11 | 16 (21.3%) | 1.37 (0.78,2.41) | 0.27 | 1 (1.0%) |
| *P_heterogeneity_*‡ |  |  | <0.001 |  |  | 0.002 |  |
| Age at diagnosis, y |  |  |  |  |  |  |  |
| 0-4 | 120 (11.8%) | 1.00 (ref.) |  | 147 (16.0%) | 1.0 (ref.) |  | 14 (1.1%) |
| 5-9 | 55 (9.4%) | 0.83 (0.55,1.26) | 0.37 | 72 (13.8%) | 0.99 (0.70,1.39) | 0.95 | 1 (0.1%) |
| 10-14 | 43 (8.3%) | 0.85 (0.52,1.38) | 0.50 | 61 (13.5%) | 1.01 (0.68,1.49) | 0.86 | 4 (0.6%) |
| *P_trend_*‡ |  |  | 0.43 |  |  | 0.88 |  |
| Decade of diagnosis |  |  |  |  |  |  |  |
| <1980 | 55 (9.1%) | 1.00 (ref.) |  | 76 (14.2%) | 1.00 (ref.) |  | 4 (0.5%) |
| 1980-1984 | 66 (10.8%) | 1.32 (0.87,2.00) | 0.19 | 101 (18.7%) | 1.49 (1.05,2.12) | 0.03 | 8 (1.0%) |
| 1985-1991 | 80 (9.9%) | 1.13 (0.67,1.88) | 0.65 | 86 (12.1%) | 0.95 (0.61,1.50) | 0.83 | 7 (0.7%) |
| *P_trend_*‡ |  |  | 0.76 |  |  | 0.58 |  |

*Adjusted for maternal age and parity; Abbreviations: CNS=Central Nervous System; NH=non-heritable; H=heritable; RR=relative risk; CI=confidence interval.

†No model was fit because of small number of still-births;

‡P-value based on two-sided Wald test

§Relates to all survivors not treated with any radiotherapy

**Supplementary Table 2.** Sensitivity analysis comparing risk of developing specific pregnancy/labour complication between survivors treated with abdominal radiotherapy to survivors not treated with radiotherapy (RT) by Hospital Episode Statistics (HES) calendar year (<2002 vs. ≥ 2002).

| **Pregnancy or labour outcome** | **HES years** | **Abdominal RT vs No RT** | **P_interaction_*** |
| --- | --- | --- | --- |
|  |  | **RR (95%CI)** |  |
| Hypertension complicating pregnancy | 1997-2001 | 1.00 (ref.) |  |
|  | 2002-2012 | 1.22 (0.60,2.31) | 0.68 |
| Gestational diabetes mellitus | 1997-2001 | 1.00 (ref.) |  |
|  | 2002-2012 | 1.41 (0.31,6.44) | 0.67 |
| Anaemia complicating pregnancy | 1997-2001 | 1.00 (ref.) |  |
|  | 2002-2012 | 0.64 (0.24,1.74) | 0.38 |
| Malpresentation of foetus | 1997-2001 | 1.00 (ref.) |  |
|  | 2002-2012 | 0.28 (0.06,1.40) | 0.12 |
| Uterine scar from previous surgery | 1997-2001 | 1.00 (ref.) |  |
|  | 2002-2012 | 0.66 (0.29,1.57) | 0.36 |
| Foetal problems | 1997-2001 | 1.00 (ref.) |  |
|  | 2002-2012 | 1.12 (0.44,2.74) | 0.83 |
| Premature rupture of membranes | 1997-2001 | 1.00 (ref.) |  |
|  | 2002-2012 | 1.77 (0.64,4.84) | 0.28 |
| Prolonged (post-term) pregnancy | 1997-2001 | 1.00 (ref.) |  |
|  | 2002-2012 | 1.50 (0.43,5.23) | 0.54 |
| Abnormalities of forces of labour | 1997-2001 | 1.00 (ref.) |  |
|  | 2002-2012 | 0.41 (0.07,2.51) | 0.34 |
| Long labour | 1997-2001 | 1.00 (ref.) |  |
|  | 2002-2012 | 2.11 (0.84,5.2) | 0.14 |
| Obstructed labour | 1997-2001 | 1.00 (ref.) |  |
|  | 2002-2012 | 3.08 (0.78,12.20) | 0.11 |
| Delivery complicated by foetal stress | 1997-2001 | 1.00 (ref.) |  |
|  | 2002-2012 | 1.20 (0.71,2.03) | 0.49 |
| umbilical cord complications | 1997-2001 | 1.00 (ref.) |  |
|  | 2002-2012 | 0.37 (0.07,1.82) | 0.22 |
| Perineal laceration | 1997-2001 | 1.00 (ref.) |  |
|  | 2002-2012 | 1.32 (0.88,1.97) | 0.18 |
| Postpartum haemorrhage | 1997-2001 | 1.00 (ref.) |  |
|  | 2002-2012 | 1.00 (0.40,2.48) | 0.98 |
| Elective Caesarean | 1997-2001 | 1.00 (ref.) |  |
|  | 2002-2012 | 1.26 (0.77,2.05) | 0.30 |
| Emergency Caesarean | 1997-2001 | 1.00 (ref.) |  |
|  | 2002-2012 | 1.03 (0.54,1.96) | 0.92 |
| Supervision of high risk pregnancy | 1997-2001 | 1.00 (ref.) |  |
|  | 2002-2012 | 0.59 (0.14,2.44) | 0.47 |
| low birth weight | 1997-2001 | 1.00 (ref.) |  |
|  | 2002-2012 | 0.82 (0.37,1.83) | 0.62 |
| Premature delivery | 1997-2001 | 1.00 (ref.) |  |
|  | 2002-2012 | 0.51 (0.22,1.17) | 0.11 |

*****P-value based on a two sided Wald test. CI=confidence interval; RR=relative risk.
